# Rare spontaneous monochorionic dizygotic twins: a case report and a systematic review

**DOI:** 10.1186/s12884-022-04866-x

**Published:** 2022-07-14

**Authors:** Giulia Trombetta, Dora Fabbro, Eliana Demori, Lorenza Driul, Giuseppe Damante, Serena Xodo

**Affiliations:** 1 Department of Gynecology and Obstetrics, School of Medicine of Udine, Udine, Italy; 2 Istituto Di Genetica Medica, Azienda Sanitaria Universitaria Friuli Centrale, Udine, Italy; 3 Dipartimento Di Area Medica, Università Degli Studi Di Udine, Udine, Italy; 4 Department of Gynecology and Obstetrics, Azienda Sanitaria Universitaria Friuli Centrale, Presidio Ospedaliero Santa Maria della Misericordia, Udine, UD Italy

**Keywords:** Monochorionic dizygotic twins, Situs inversus, Biliary atresia splenic malformation

## Abstract

**Background:**

Monochorionic dizygotic twins are a rare condition, mostly related to assisted reproductive technology. This type of twinning is burdened by the same risk of pregnancy complications found in monochorionic monozygotic pregnancies.

**Case presentation:**

We report a case of spontaneous monochorionic dizygotic twins sharing situs inversus abdominalis and isolated levocardia, with only one twin affected by biliary atresia with splenic malformation syndrome. We also conducted a literature review of the 14 available documented monochorionic dizygotic twin gestations spontaneously conceived.

**Conclusions:**

It is still unclear how this unusual type of twinning can occur in spontaneous conception. The evidence so far suggest the importance to timely diagnose the chorionicity, in order to adequately manage the typical complications associated with monochorionicity.

**Supplementary Information:**

The online version contains supplementary material available at 10.1186/s12884-022-04866-x.

## Background

Monochorionic dizygotic (MCDZ) twins are a rare condition, mostly related to assisted reproductive technology (ART) [1]. Here we present a case of spontaneous monochorionic dizygotic twins with isolated levocardia and a normal cardiac structure, situs inversus abdominalis, discordant for abdominal anomalies, with only one twin affected by biliary atresia with splenic malformation syndrome (BASM).

We reviewed all cases of spontaneous monochorionic dizygotic twins reported in literature, highlighting the clinical features, the obstetrical implications and the challenges related to this unusual and still not fully known twinning event.

## Case presentation

A 27-year-old woman, gravida 2, para 0, with no history of previous disease and no family history of congenital anomalies, smoker and with a BMI of 18.3 received a diagnosis of spontaneous twin pregnancy at 12 weeks. The assessment of chorionicity in this gestational age was however hampered by the presence of an hematoma measuring 44 × 38 mm separating the two membranes, thus preventing the identification of the “lambda” or “T” sign.

**Fig. 1:**
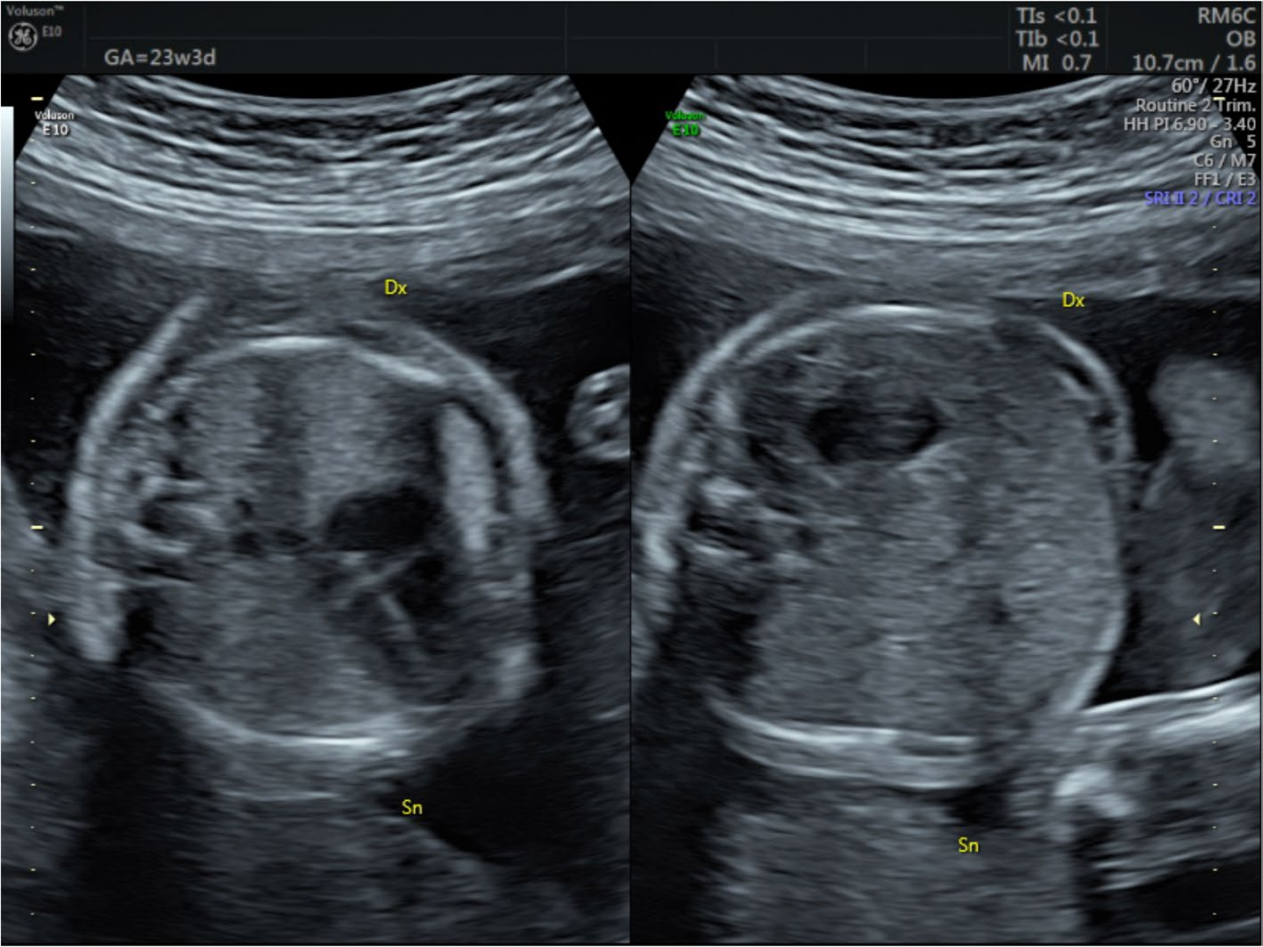
Situs inversus abdominalis with isolated levocardia of one twin at anomaly scan: stomach lies on the right, whereas the cardiac apex is pointing toward the left

The anatomy scan at 20 weeks revealed two female fetuses, both presenting isolated levocardia (IL) with normal heart and situs inversus abdominalis with a left-sided liver and right-sided stomach and spleen (Fig. 1). Noteworthy, in one fetus (A) the gallbladder was not visible.

An amniocentesis was performed, with conventional cytogenetic evaluation indicating normal karyotype 46XX for both fetuses. No genetic anomaly was reported with the Chromosomal Microarray Analysis (CMA). Zygosity was assessed by microsatellite analysis; as shown in Table 1, twins share only a fraction of paternal and maternal alleles, indicating dizygosity.

**Table 1:** Microsatellites analysis of twins and parents. Numbers indicate alleles

LOCUS	Mother	TWIN 1	TWIN 2	Father
D10S1248	12/15	12/14	12/12	12/14
vWA	16/17	17/17	17/17	17/17
D16S539	10/11	11/12	10/12	11/12
D2S1338	18/19	19/25	19/20	20/25
Amelogenin	XX	XX	XX	XY
D8S1179	12/16	12/15	12/14	14/15
D21S11	29/30	29/30	28/30	28/30
D18S51	12/19	17/19	13.2/19	13.2/17
D22S1045	16/16	15/16	16/18	15/18
D19S433	12/15.2	12/15.2	12/12	12/16
TH01	9.3/9.3	8/9.3	7/9.3	7/8
FGA	20/25	20/24	24/25	23/24
D2S441	11/14	11/14	11/11	11/11
D3S1358	14/17	14/14	14/17	14/17
D1S1656	15/15.3	15/15.3	15/15.3	15/17.3
D12S391	17.3/20	18/20	18/20	18/18.3
SE33	15/30.2	18/30.2	15/18	18/26.2

At 36 weeks one fetus was diagnosed to be growth restricted, having an abdominal circumference and an estimated fetal weight less than 3rd centile. At 37 weeks and 1 day, the pulsatility index (PI) of the umbilical artery of the growth restricted fetus, with an estimated fetal weight of 2171 g (below the 3rd centile, according to Hadlock growth chart), was 0.89 (corresponding to 46° centile), while the PI of the middle cerebral artery was 1.45 (26° centile). By contrast, the other twin had an estimated fetal weight of 2521 g with normal Doppler parameters. At 37 weeks and 3 days the mother underwent a scheduled cesarean delivery. The birthweight of the two female neonates were 2430 g and 2185 g.

**Fig. 2:**
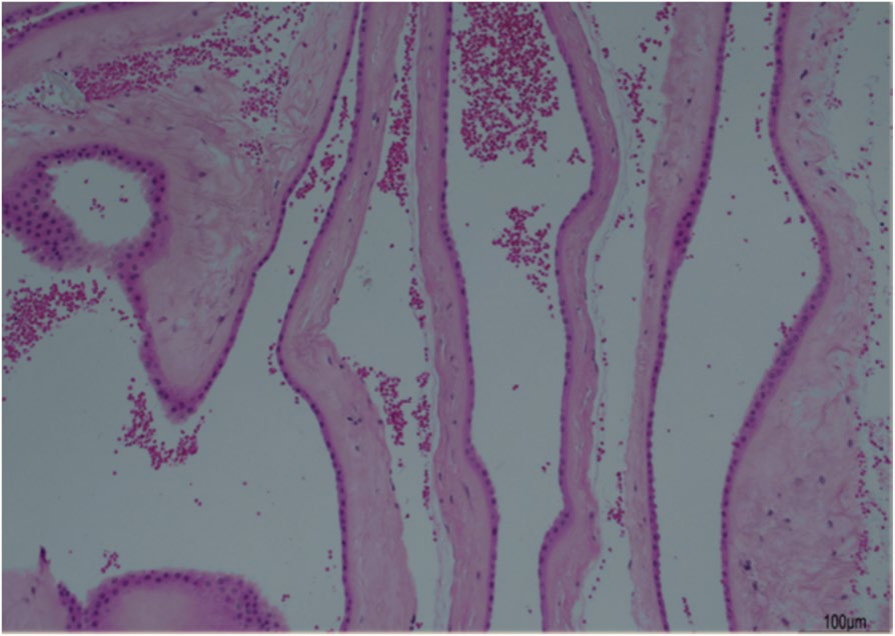
Cross-section through the placental septal membrane roll demonstrates a septum with two layers of amnion, without intervening central chorion (Hematoxillin-Eosin image obtained using ECLPISE Ni-U equipment, with 10 × magnification, acquired through DS-Fi3 Nikon Digital Camera)

The histological analysis of the placenta confirmed the monochorionicity of the twin pregnancy, revealing the juxtaposition of an amnion on each surface of the dividing membrane (Fig. 2: Hematoxillin-Eosin image obtained using ECLPISE Ni-U equipment, with 10 × magnification, acquired through DS-Fi3 Nikon Digital Camera). The chorion was not visible between the two amnion surfaces, thus excluding the rare occurrence of partially fused placentas [2, 3]. Postnatally, by using DNA extracted from blood samples, the molecular analysis confirmed the dizygosity of the twins, as already previously determined through amniocentesis.

Moreover, the two babies turned out to have the same situs anomaly, with IL and situs inversus, thus confirming the prenatal diagnosis. In order to identify possible genetic causes of such a concordant abnormal phenotype, the twins were subjected to clinical exome analysis with evaluation of 17 genes known to be associated to situs inversus. However, according to the variant classification ACMG (The American College of Medical Genetics and Genomics), no pathogenic or probably pathogenetic variants have been identified. By opening the analysis to the whole clinical exome (4490 genes), the twins did not share pathogenic or probably pathogenetic variants. In addition, the twin whose gallbladder was not visualized prenatally, developed jaundice with acholic stool in her neonatal period.

**Fig. 3:**
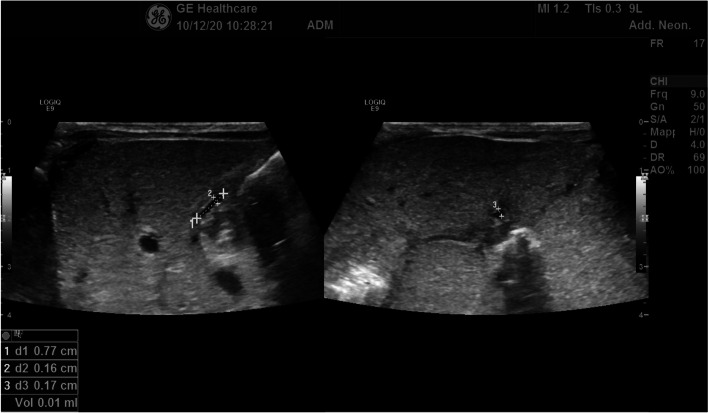
Abdominal ultrasound at 35 days of extrauterine life of the twin with jaundice: in the liver, a small and atretic gallbladder, not expanding after 3 h fasting, confirmed suspicion of biliary atresia

The complete abdominal scan performed at 20 days of extrauterine life showed a left sided, damage-free liver with regular size (lateral diameter of 6,3 cm), a reversed relation between superior mesenteric vein and artery and right-placed inferior vena cava in relation to the aorta. These findings perfectly fit with abdominalis situs inversus with isolated levocardia. The scan revealed for the first time a shriveled gallbladder, which led to the diagnosis of biliary atresia type IV associated with splenic malformations syndrome (Fig. 3). The clinical exome analysis was unable to identify the genetic cause of these abdominal abnormalities.

The twin with BASM at 38 days of extrauterine life underwent Kasai portoenterostomy, second-hand appendectomy and Ladd bridle dissection. Later, during the fifth month of extrauterine life, the baby was diagnosed to have a subclinical acute cholangitis, which was treated with continuous infusion of piperacillin tazobactam during hospitalization. Unfortunately, the baby had recurrent cholangitis during the whole first year of extrauterine life, and these are still occurring.

## Discussion and conclusions

A systematic review was conducted using Pubmed, Scopus, OVID, and Cochrane Library electronic databases. The citations were identified with the use of a combination of the following key words: “monochorionic dizygotic twins”; “monochorionic dizygotic chimerism”; “monochorionic dizygotic freemartinism”; “spontaneous monochorionic dizygotic twins”; “spontaneous monochorionic heterosexual twins” from the inception of each database through January 2022. Only articles in English language were selected, while no restrictions for geographic location were applied (Supplementary material).

Overall 14 cases of spontaneously conceived and 4 cases of monochorionic dizygotic twins conceived after ovulation induction were identified. All available articles were case reports. A description of each case is provided in two tables: Table 2 describes case reports on spontaneously conceived monochorionic dizygotic twins [4–17], while Table 3 describes case reports on monochorionic dizygotic twins conceived after ovulation induction [18–21]. The mean maternal age at delivery in the group of spontaneously conceived monochorionic dizygotic twins was 31.08 weeks (SD ± 4.69). The sonographic assessment of chorionicity was established in the first trimester in 11 cases [5, 6, 8–16]. Follow-up scans revealed the following complications during pregnancy: 2 cases of Twin-to-Twin Transfusion syndrome (TTTS) [5, 14],  1 case of Twin Reversed Arterial Perfusion sequence (TRAP) [7], 1 case of Twin Anemia Polycythemia sequence (TAPS) [13] and 1 case of discordant growth pattern [10]. Sex discordance between twins in monochorionic pregnancy was reported in 4 cases [12, 13, 15, 16]. Amniocentesis was performed to validate dizygosity in 2 cases [12, 15], while in 1 case it was done to exclude trisomy 21 [6]. Out of 14 cases reported, 1 case underwent miscarriage after laser procedure performed at 18 weeks gestation for TTTS [5], 1 case underwent a voluntary termination of pregnancy because of the diagnosis of trisomy 21 in one twin [6], 6 cases had a cesarean delivery which occurred at a mean gestational age of 35.83 weeks (SD ± 2.31) [9, 11, 12, 14–16], 3 cases had a spontaneous vaginal delivery at a mean gestational age of 35 weeks (SD ± 2.64) [7, 8, 10] and 3 cases did not report the delivery mode [4, 13, 17]. A monochorionic pregnancy was confirmed at gross examination in all cases. Dizygosity was demonstrated by the phenotypical evidence of sex discordance between twins in 2 cases [4, 13] and it was proven by cytogenetic analysis showing sex discordant karyotypes between twins in 3 cases [5, 7, 8]. Chimerism was found to be confined to blood in 6 cases [6, 8, 10, 14, 16, 17], to be present in a non-shared tissue in 3 cases [9, 12, 15] and it was detected in blood as well as in tissue in 1 case [11]. The mean maternal age at delivery in the group of monochorionic dizygotic twins conceived after ovulation induction was 31.00 weeks (SD ± 3.36) [18–21]. The sonographic assessment of chorionicity was determined in the first trimester in all cases. No typical monochorionic complications were detected during the follow up scans. However, TAPS was found in one case at delivery [21]. Sex discordance between twins in monochorionic pregnancy was reported in 3 cases [18, 20, 21]. Invasive prenatal diagnosis aiming at validating dizygosity was performed in only one case [18]. Preterm delivery occurred in all cases; twins were delivered through cesarean section in 2 cases [19, 20]. A monochorionic pregnancy was confirmed at gross examination in all cases. Chimerism was found to be confined to blood in 2 cases [19, 20], to be present in a non-shared tissue in 1 case [18], and it was detected in blood as well as in tissue in 1 case [21]. In this review, we present a case of spontaneous monochorionic dizygotic twins with isolated levocardia and a normal cardiac structure, situs inversus abdominalis, discordant for abdominal anomalies, with only one twin affected by BASM. Moreover, we reviewed 14 cases of spontaneously conceived MCDZ twins [4–17]. Monochorionicity has been traditionally considered to be a guarantee of monozygosity. A growing body of evidence has now demonstrated that monochorionic twins could be dizygotic. A very rare phenomenon of “sesquizygosis” has been described in the literature [12]. In this case, the twins share the same haploid genome from one parent and, therefore, are intermediate between mono and dizygotic twinning. The microsatellite analysis of our twins excluded sesquizygosis (Table 1). A previous systematic review on this issue showed that assisted reproductive technology is the major responsible for the origin of this unusual way of twinning [1]. Several hypotheses have been advanced to explain a monochorionc dizygotic twin pregnancy: the fusion of the trophoblasts from two different embryos before implantation [22], the presence of binovular follicles where a single zona pellucida includes two distinct oocytes leading to close contact between embryos [19] and the penetration of an oocyte and second polar body surrounded by one zona pellucida by more than one sperm [23]. The chance of cell fusion seems to be small in a natural pregnancy, but not impossible to occur as proven by the number of cases found in our systematic research. Based on this review, this type of twinning is burdened by the same risk of pregnancy complications found in MCMZ pregnancies, i.e. TTTS, TRAP, TAPS and selective Fetal Growth Restriction (sFGR). These findings suggest the importance of a correct diagnosis of chorionicity in the first trimester of pregnancy, in order to timely and adequately manage possible complications. Chorionicity should be determined before 14 weeks of gestation, examining the dividing membrane carefully. In dichorionic diamniotic (DCDA) twin pregnancy, the twins are separated by a thick layer of fused chorionic membranes with two thin amniotic layers, one on each side (the so-called “full lambda” sign), while in monochorionic diamniotic (MCDA) pregnancy only two thin amniotic layers separate the two fetuses (the T-sign). In this case report the ultrasonographic examination of these signs was prevented by the presence of a large hematoma at the site of insertion of the amniotic membrane into the placenta. Since 3% of monochorionic pregnancies have two placental masses (also defined “bipartite placenta” [24]) on ultrasound and dichorionic placentae are commonly appearing as a single mass, the reliability of the number of placental masses at ultrasound is questionable. Therefore, we did not consider this feature for the diagnosis of chorionicity. Interestingly, bipartite placenta seems to have some relevant clinical implications. A recent study found bipartite placenta in five MCDA pregnancies and showed that this occurrence was associated with a higher rate of complications, such as TTTS and sFGR and might impair prenatal surgical interventions [24]. The diagnosis of monochorionicity is even more important in the context described in our case report, since sex discordance of twins could lead to the automatic assumption of dichorionicity. Chimerism is characterized by cells originating from more than one genetically distinct zygote. Chimerism was found in most reviewed cases. Blood confined chimerism is likely to be consequent to the blood sharing between the dizygotic twins via the unique placenta. It has been theorized that the “outer cell mass” of the two distinct dizygotic embryos undergo fusion with the development of a single chorion and anastomoses. However, in 21.42% of cases tissue chimerism was found, which is more difficult to explain [9, 11, 15]. It is unknown whether chimerism has clinical consequences. Bogdanova et al. reported a possible case of human freemartinism in a female twin with aplasia of the uterus [25]. According to the Author the lack of Mullerian structures in this female was caused by her exposition to the effect of the Mullerian inhibiting substance transferred from the male twin via the common placenta in early pregnancy. Recently, Peters et al. investigated whether there is a prevalence of male microchimerism in women with Mayer–Rokitansky–Küster–Hauser (MRKH) syndrome [26]. However, their observational case–control study, involving 95 women with MRKH syndrome and 99 control women, showed that the prevalence of male microchimerism was significantly higher in the control group than in the MRKH group, thus rejecting the initial hypothesis. We compared our case report with the cases of spontaneously conceived MCDZ twins present in our systematic review. Molecular analysis performed on tissue and blood samples of the two female twins confirmed dizygosity. What is peculiar of our case report, is the very rare anomaly shared by both twins, though in a different way. Both have IL and situs inversus abdominalis, but only one twin is affected by biliary atresia with splenic malformation syndrome. No genetic cause of these abnormalities was identified by clinical exome analysis; but this should not rule out genetic determinants of the phenotypic abnormalities. There are indeed several distinct methodological features able to explain the non-identification of genetic causes. Among them, the fact that the clinical exome evaluates only genes known to be associated to human diseases or the possibility that the putative causative variant might be located in control regions of gene expression (promoter or enhancers, for example), not analyzed by the exome approach. Biliary atresia is recognized as a key feature in two distinct types of syndromes: the Cat-eye syndrome (CES) and the Biliary Atresia Splenic Malformation (BASM) syndrome. The first one is determined by aneuploidy of chromosome 22 and patients affected typically have coloboma, cardiac anomalies and anorectal malformations. On the other hand, BASM is characterized by a constellation of visceral anomalies in different combinations. Patients with BASM could have polysplenia, asplenia or double spleen, situs inversus with and without malrotation; preduodenal portal vein, a complete absence of intrahepatic vena cava and cardiac anomalies. According to some researchers [27, 28] the association between BA and laterality defects of the abdominal viscera may suggest a defect in the embryonic development to explain the etiology of BASM. The bile duct development begins at 4 gestational weeks and ends at about 13 weeks; in the same period laterality defects, such as development of left–right axis reversal (GA 2–3 weeks), splenic malformations (GA 3–6 weeks), preduodenal portal vein (GA 4–8 weeks), and interrupted vena cava (GA 6–8 weeks) are thought to occur [27]. A recent metanalysis, aiming at analyzing the characteristics of biliary atresia in twins, found that 97% of twins were discordant for the anomaly. In more than half of the cases twins were monozygotic, thus indicating that zygosity is not the main causative factor of the onset of the disease [29]. However, even assuming the role of epigenetic factors in the pathogenesis of BA, our case report still remains a fascinating enigma. It is hard to find an explanation of why two monochorionic dizygotic sex concordant twins should share a very rare laterality anomaly of the abdominal viscera, sparing one twin from developing biliary atresia. The main strength of this study is the singularity and originality of our case report, where two MCDZ twins share the same malformation in a slight different way, with important clinical consequences on one twin. Moreover, we did a comprehensive systematic review on MCDZ pregnancies naturally conceived, which is the first in the literature to the best of our knowledge. It is interesting to note that when spontaneously conceived MCDZ pregnancies were affected by a malformation, this was present in one twin only, according to our review. Our case report is therefore unique. However, some limitations should be recognized too. First of all, we did not perform the whole exome sequencing, thus preventing the possibility to identify some variants located in control region of gene expression. In addition we did not investigate the occurrence of chimerism, either in blood and in other different tissues.

**Table 2:** Case reports of spontaneously conceived monochorionic dizygotic twins

Author	Publication year	Maternal age	US Chorionicity	US follow up	Invasive prenatal test	Outcome pregnancy	Gross examination of placenta	Postnatal test	Conclusion
Nylander [4]	1970	35	–	–	–	Delivery of heterosexual monochorionic twins: Twin A male, weighing 5 lb and 8 oz; Twin B female: weighing 3 lb and 14 oz	MC placenta	–	First case of MCDZ twins
Quintero [5]	2003	28	MC-DA (I trimester)	18 w: stage I TTTS Laser therapy + reductive amniocentesis	–	miscarriage	MC placenta	Cytogenetic analysis: -Recipient twin: 46 XX karyotype -Donor twin: 46 XY karyotype Molecular analysis using multiple polymorphic microsatellites confirmed dizygosity	First case of MCDZ pregnancy complicated by TTTS
Shalev [6]	2006	39	MC-DA (I and II trimesters)	-	AMNIOCENTESIS for maternal age (-normal male karyotype -47 XY + 21) CORDOCENTESIS Blood chimerism	Termination of pregnancy: -normal fetus -abortus with physical features consistent with Down syndrome	MC placenta		Blood chimerism in MC DZ twins
Lattanzi [7]	2006	33	-	27 w: TRAP s (- inhomogeneus mass without cardiac activity, containing large cystic spaces and no other recognizable visceral structures; -the other fetus: normal)	Not done	SVD at 32 w: -first baby: male, weighing 1850 gr, non external malformations -second twin:33 × 40 cm globular mass of tissue, coated by skin and hairs and hair glands, weighing 1070 gr, without upper or lower limbs	MC placenta	-peripheral blood: normal male karyotype in first twin -skin biopsy and inner mass connective tissue: homogeneous 46 XX karyotype	Sex discordance bw MC twins
Hackmon [8]	2009	33	MC-DA at 12.6 w	–	Not done	SVD at 37 w	MC placenta	-buccal cell DNA: normal 46 XX karyotype in the girl and normal 46 XY in the boy -lymphocyte DNA: 46 XX [26]/46 XY [24] in the girl and 46 XY [25]/46 XX [25] in the boy	Sex discordance bw MC twins Blood chimerism
Umstad [9]	2012	36	MC-DA (12 w)	–	Not done	TTTS stage I Quintero CD at 36 w after CCS	MC placenta	-Buccal cell DNA: 3 loci shared by the twins -Leukocyte DNA: 12 of 12 loci shared by the twins	MC Twins DZ in a nonshared tissue, apparently MZ in blood
Kanda [10]	2013	28	MC-DA (I trimester)	Discordant growth pattern	Not done	SVD at 36 w: -twin 1: phenotypically normal, boy, birthweight 2944 gr -twin 2: phenotypically normal, boy, birthweight 2502 gr	MC placenta	At 1 month of age: -blood karyotype (from lymphocytes): 46 XX [5]/ 46 XY [25] in the boy; 46 XY [17]/ 46 XX [13] in the girl At 6 months of age: -skin fibroblasts karyotype: normal for both twins	Blood chimerism
Rodriguez –Buritica [11]	2014	21	MC-DA	–	–	CD at 39 w: -male twin 2300 gr (at 2 months of age: critical aortic stenosis and glanular hypospadias) -girl 2100 gr	MC placenta	Male twin: -blood karyotype: 46 XY [14]/ 46 XX [16] -skin karyotype: 46 XY [18]/ 46 XX [12] -blood interphase FISH X/Y analysis: 46 XY in 43% 46 XX in 57% Female twin: -blood karyotype: 46 XX [9]/ 46 XX [21] -skin karyotype: 46 XX [32] -blood interphase FISH X/Y analysis: 46 XX in 44.5% 46 XY in 55.5%	Blood and tissue (skin) chimerism
Gabbett [12]	2019	28	MC-DA (I trimester)	Follow up scans: sex discordance bw twins	AMNIOCENTESIS to validate zygosity: 46 XX/ 46 XY chimerism in each twin	CD at 33 w: Twin1 phenotypically male Twin 2 phenotypically female	MC placenta	Twin 2: At 4 w of age a below shoulder amputation due to right brachial artery thromboembolism At 3 y of age: prophylactic oophorectomy due to gonadal dysgenesis	Sesquizygotic twinning
K. Chen [13]	2020	32	MC-DA at 14 w	Follow up scans: sex discordance bw twins At 28 + 6 TAPS	Not done	Delivery at 31 + 2 w (following CCS and Magnesium sulfate); TAPS confirmed after birth: -Hb 25.2 g/dL in recipient twin -Hb 7.5 g/dL in donor twin Sex discordance confirmed post-natally	MC placenta	Not done	First case of TAPS in MC DZ twins diagnosed prenatally
Armitage [14]	2020	–	MC-DA (I trimester	Follow up scans: TTTS from II trimester	–	CD at 35 w: 2 female twins	MC placenta	Twin A: bilateral retinoblastoma at 7 months of age Twin B: unaffected Test on peripheral blood and skin biopsy In both twins: -Twin A harbored the RBP1 pathogenetic variant in the skin and the blood -Twin B displayed the RBP1 pathogenetic variant in blood only	Blood confined chimerism
Daum [15]	2020	30	MC-DA	NT: normal Anomaly scan at 17 w: female and male fetuses without malformations	AMNIOCENTESIS to validate zygosity: Dizygotic twins, female and male, with normal karyotypes	CD at 34 w because of TAPS: -girl, weighing 2150 gr, with Hb 21.8 g/dL -boy, weighing 2130 gr, with Hb 10.2 g/dL	MC placenta	Cord blood karyotype: -male: chimerism with 17% 46 XX -female: chimerism with 27% 46 XY Buccal smear FISH: -male: no chimerism -female: 1% chimerism 2 y follow up buccal smear FISH: -male: 3% chimerism (46 XX) -female: 5% chimerism (46 XY)	Non confined blood chimerism
Yoshida [16]	2021	30	MC-DA (I trimester)	Follow up scans: sex discordance bw twins	–	CD at 38 w Twin A: phenotypically female newborn, weighing 2612 gr; Twin B: phenotypically male newborn, weighing 2458 gr	MC placenta	Karyotyping on umbilical cord: chimeric karyotypes Twin A 46,XX [15]/46,XY [15] Twin B 46,XY [21]/46,XX [9] At 7 months of age karyotyping performed from blood samples: chimerich karyotypes: Twin A 46,XY [7]/46,XX [13] Twin B 46,XX [9]/46, XY [11] At 1 year-old karyotyping from buccal swab cells: normal female and normal male karyotypes: Twin A 46,XX[98]/46,XY [2] At 3 years of age: blood group: Twins with B/0 chimera	Sex discordant MC DZ twins with chimeric blood group types
J. Chen [17]	2021	–	–	–	–	–	MC placenta	27 years after birth Peripheral blood Buccal cells Twin 1 (male, proband’s brother): has the same DNA in different tissues Twin 2 (female, proband): 2 sets of DNA in her blood with 92.84 + 1.80% chimerism; no chimerism in oral mucosa or endometrium	Blood confined chimerism

**Table 3:** Case reports of monochorionic dizygotic twins conceived after ovulation induction

Author	Publication year	Maternal age	US chorionicity	US follow up	Invasive prenatal test	Outcome pregnancy	Gross examination of placenta	Postnatal test	Conclusion
Ginsberg [18]	2005	35	MC-DA (I trimester)	US 20 w: discordant sex	Chorionic villi sampling at 11 wk from two different placental areas: 46, XY for both fetuses Amniocentesis after US at 20wks: Twin A 46,XY; Twin B, 46 XX FISH on AF samples: 4% of the cells demonstrating chr Y in Twin B, diagnosis of Dizygosity.	Delivery 22wks, expired Normal anatomy both twins at the autopsy	MC placenta	–	MCDZ twins AF chimerism?
Aoki [19]	2006	27	MC-DA (I trimester)	–	–	CD at 34 w -Twin A, male, birthweight 2002 g. Blood group AB -Twin B, male, birthweight 2132 g. Blood group B	MC placenta	Chimerism blood group: -Twin A: AB (88%)/B(12%) -Twin B: B (99%)/AB (1%) DNA polymorphism analysis: 5 loci/9 loci different in peripheral lymphocytes and hair root cells. No chimerism.	Confined blood chimerism in MC-DZ same gender twins
Mayeur le bras [20]	2016	32	MC-DA (I trimester)	US 18 + 4 w: Twin A female, Twin B male. Hospitalization at 25w for preterm labor: CCS prophylaxis, tocolysis.	Not performed (refused by the patient)	pPROM at 36 + 1 w: CD because of umbilical prolapse Twin B. Twin A: female 2000 g; Twin B: male, birthweight 1970 g Normal genitalia in both twin	MC placenta A-V and A-A anastomoses	Blood chimerism No chimerism on buccal cells. Cord blood chromosome analysis at birth: -Twin A (girl) 46,XX(11)/46,XY(4) -Twin B (boy) 46,XY(7)/46,XX(8) Blood chromosome analysis at 5 months: -Twin A (girl) 46,XX(8)/46,XY(7) -Twin B (boy) 46,XY(11)/46,XX(5)	Confined blood chimerism in MC DZ twins
Suzuki [21]	2019	30	MC-DA (I trimester)	Follow up: normal growth, no differences in amniotic fluid	–	SVD at 33 + 1w TAPS found on the neonates -Male twin: birthweight 1919 gr, polycythemia (Hb 23 g/dL) -Female twin: birthweight 1990 gr, anemia (Hb 8.6 g/dL)	MC placenta	Blood chimerism analyzed with FISH: - 46% of the male’s lymphocytes XX karyotype - 36.6% of the female’s lymphocytes XY karyotype Buccal mucosa: - male: 99 XY cells, 1 XX cell - female: 98 XX cells, 2 XY cells	MC twins with TAPS

In conclusion, spontaneously conceived MC-DZ twins are a rare condition, with only 14 cases described in literature. The evidence so far suggests the importance to timely diagnose the chorionicity, in order to adequately manage the typical complications associated with monochorionicity. Furthermore, the clinician should keep in mind that monochorionicity does not always correspond to monozygosity. Rarely, MC twins could be DZ, even in naturally conceived pregnancies, as shown in this comprehensive review. It is still unclear how this unusual type of twinning can occur in spontaneous conception. The mystery deepens considering the peculiarity of our case report, where the two MCDZ twins share a very rare anomaly, for which no genetic cause has been found through clinical exome analysis.

## Supplementary Information

**Additional file 1: Supplementary figure S1.  **Flow diagram of inclusion of articles.

## Data Availability

The datasets used and/or analysed during the current study are available from the corresponding author on reasonable request.

## Abbreviations

MC-DZ: Monochorionic dizygotic
ART: Assisted reproductive technology
BASM: Biliary atresia with splenic malformation syndrome
IL: Isolated levocardia
CMA: Chromosomal Microarray Analysis
PI: Pulsatility index
ACMG: The American College of Medical Genetics and Genomics
TTTS: Twin-to-Twin Transfusion syndrome
TRAP: Twin Reversed Arterial Perfusion sequence
TAPS: Twin Anemia Polycythemia sequence
MCMZ: Monochorionic monozygotic
sFGR: Selective fetal growth restriction
DCDA: Dichorionic diamniotic
MCDA: Monochorionic diamniotic
MRKH: Mayer–Rokitansky–Küster–Hauser
CES: Cat-eye syndrome
BA: Biliary atresia
