# Supplementary material for: Rare spontaneous monochorionic dizygotic twins: a case report and a systematic review
Source: BMC Pregnancy Childbirth. 2022 Jul 14;22:564. doi: 10.1186/s12884-022-04866-x (PMC9284757; doi:10.1186/s12884-022-04866-x)

Rare spontaneous monochorionic dizygotic twins: a case report and a systematic review.

January 14th 2022

Pubmed

354 articles

(monochorionic AND dizygotic AND twins; OR monochorionic AND dizygotic AND chimerism; OR monochorionic AND dizygotic AND freemartinism; OR monochorionic AND dizygotic AND spontaneous; OR monochorionic AND heterosexual AND spontaneous)

Scopus

367 articles

(monochorionic AND dizygotic AND twins; OR monochorionic AND dizygotic AND chimerism; OR monochorionic AND dizygotic AND freemartinism; OR monochorionic AND dizygotic AND spontaneous, OR monochorionic AND heterosexual AND spontaneous)

Ovid

62 articles

(monochorionic AND dizygotic AND twins; OR monochorionic AND dizygotic AND chimerism; OR monochorionic AND dizygotic AND freemartinism; OR monochorionic AND dizygotic AND spontaneous, OR monochorionic AND heterosexual AND spontaneous)

Supplementary figure S1 – *Flow diagram of inclusion of articles*

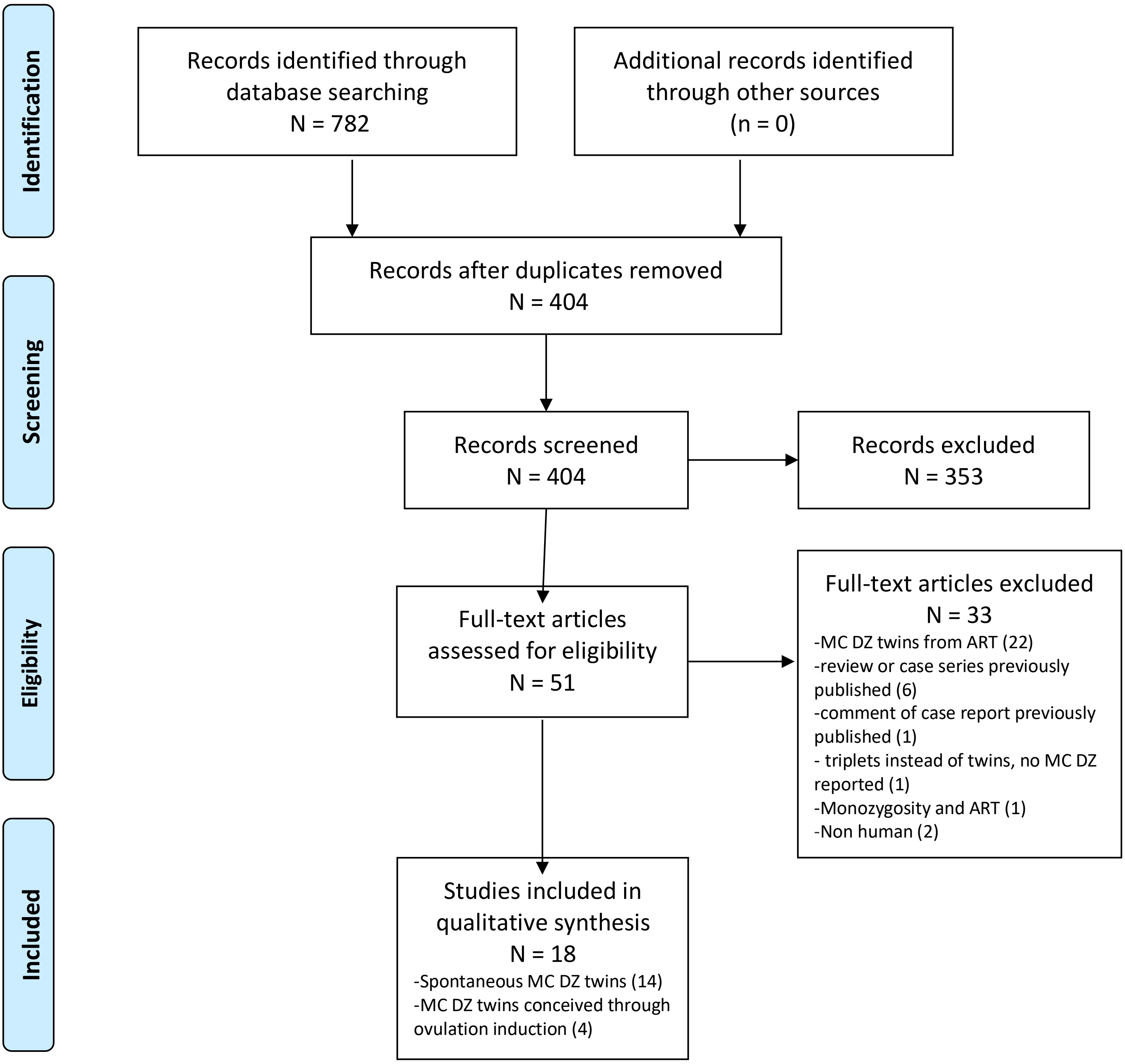

Supplement: Supplementary file 1 — Additional file 1: Supplementary figure S1. Flow diagram of inclusion of articles. [file 12884_2022_4866_MOESM1_ESM.pdf]
